# Preamble Injection-Based Jamming Method for UAV LoRa Communication Links

**DOI:** 10.3390/s26020614

**Published:** 2026-01-16

**Authors:** Teng Wu, Runze Mao, Yan Du, Quan Zhu, Shengjun Wei, Changzhen Hu

**Affiliations:** 1 School of Cyberspace Science and Technology, Beijing Institute of Technology, Beijing 100081, China; 3220205106@bit.edu.cn (T.W.); 3120205527@bit.edu.cn (R.M.); 3220215217@bit.edu.cn (Y.D.); 3220245307@bit.edu.cn (Q.Z.); chzhoo@bit.edu.cn (C.H.); 2 Beijing Key Laboratory of Software Security Engineering Technology, Beijing Institute of Technology, Beijing 100081, China

**Keywords:** UAV communication, LoRa communication, preamble injection, communication security, information confrontation

## Abstract

The widespread use of low-cost, highly maneuverable unmanned aerial vehicles (UAVs), such as racing drones, has raised numerous security concerns. These UAVs commonly employ LoRa (Long Range) communication protocols, which feature long-range transmission and strong anti-interference capabilities. However, traditional countermeasure techniques targeting LoRa-based links often suffer from delayed response, poor adaptability, and high power consumption. To address these challenges, this study first leverages neural networks to achieve efficient detection and reverse extraction of key parameters from LoRa signals in complex electromagnetic environments. Subsequently, a continuous preamble injection jamming method is designed based on the extracted target signal parameters. By protocol-level injection, this method disrupts the synchronization and demodulation processes of UAV communication links, significantly enhancing jamming efficiency while reducing energy consumption. Experimental results demonstrate that, compared with conventional approaches, the proposed continuous preamble injection jamming method achieves improved signal detection accuracy, jamming energy efficiency, and effective range. To the best of our knowledge, this protocol-aware scheme, which integrates neural network-based signal perception and denoising, offers a promising and cost-effective technical pathway for UAV countermeasures.

## 1. Introduction

The rapid proliferation of low-cost, highly maneuverable unmanned aerial vehicles (UAVs), such as racing drones, has significantly expanded the scope of UAV applications in aerial photography, entertainment, competitive events, and security surveillance. However, the widespread adoption of these platforms has also raised serious concerns regarding low-altitude safety, privacy breaches, and unauthorized intrusions, posing substantial challenges to public security and information protection [1].

To ensure reliable remote control and telemetry, UAVs commonly utilize the LoRa (Long Range) communication protocol, which is characterized by long-range transmission, low power consumption, and robust anti-interference capabilities. Despite these advantages, traditional countermeasure techniques against LoRa-based UAV communication links—including wideband noise jamming and signal blocking—often suffer from delayed response, poor adaptability, high energy consumption, and significant collateral impact on legitimate communications. These limitations hinder effective and precise interference in dynamic and complex electromagnetic environments.

Recent years have seen increasing research on UAV countermeasures leveraging LoRa communications, with various jamming techniques proposed to disrupt or manipulate UAV control and telemetry links. For instance, predictive jamming methods [2] attempt to anticipate and block LoRa transmissions by analyzing communication patterns and timing, thereby increasing the likelihood of successful interference. Replay jamming approaches [3] involve capturing legitimate LoRa signals and retransmitting them to confuse or destabilize UAV systems, often targeting authentication and synchronization procedures. Selective inference jamming [4] focuses on identifying and targeting specific control or data packets, enabling more efficient and targeted disruption of UAV operations while minimizing overall energy consumption. While these methods demonstrate the feasibility of LoRa-based UAV countermeasures, they often face limitations in terms of adaptability, precision, or resource efficiency.

Recent advances in artificial intelligence and wireless signal processing have introduced new opportunities for intelligent and efficient communication countermeasures [5]. In particular, deep learning-based signal recognition and parameter extraction methods enable accurate identification of protocol features and physical layer parameters in target UAV links, thus facilitating targeted and high-efficiency jamming [6,7,8]. Notably, the preamble field in the LoRa protocol plays a critical role in link establishment and synchronization, directly affecting packet recognition and demodulation. Protocol-level interference mechanisms based on preamble injection offer enhanced specificity and energy efficiency compared to conventional physical-layer approaches [9,10].

This study addresses the urgent need for adaptive and efficient UAV communication jamming by proposing a novel method that combines neural network-based LoRa signal detection and reverse parameter extraction with a protocol-level preamble injection jamming method. The proposed approach leverages convolutional neural networks (CNN) and generative adversarial networks (GAN) to achieve robust signal detection and denoising in complex environments [11], thereby enabling precise interference signal generation. Experimental validation demonstrates that the preamble injection jamming method significantly improves detection accuracy, jamming efficiency, and operational range, providing a cost-effective and practical solution for UAV countermeasures in low-altitude security scenarios [6,12].

The remainder of this paper is organized as follows: Section 2 reviews related work. Section 3 presents the methods for LoRa signal acquisition and interference. Experimental procedures and analysis are discussed in detail in Section 4. Finally, Section 5 concludes the paper.

## 2. Related Works

LoRa communication technology has become a mainstream choice for UAV remote control and telemetry due to its long-range capability, low power consumption, and strong anti-interference properties [1,11]. Existing countermeasures against LoRa-based links primarily focus on physical-layer techniques such as wideband noise jamming and signal blocking. However, these methods are limited by high power requirements, slow response times, and poor adaptability to dynamic environments, often resulting in undesirable interference with legitimate communications [13,14].

Aras et al. [14] analyzed the inherent vulnerabilities of the LoRa physical layer, highlighting the extended communication window (0.9–1.5 s) afforded by chirp spread spectrum (CSS) modulation, which increases susceptibility to jamming attacks. Mikhaylov et al. [15] proposed energy depletion attacks on LoRaWAN networks by injecting forged downlink packets during the device receive window, thereby forcing extended listening periods and significantly increasing energy consumption. Ingham et al. [2] investigated predictive jamming models based on device data generation patterns, demonstrating that attackers can exploit metadata such as time slots, channels, and counters to anticipate device behavior and execute targeted signal interference.

While these studies have advanced the understanding of LoRa protocol vulnerabilities and attack methodologies, most existing approaches suffer from limited jamming effectiveness and restricted operational range [16], making them less suitable for practical UAV countermeasure scenarios. Furthermore, conventional signal detection and denoising techniques—such as wavelet transform [17,18] and empirical mode decomposition (EMD) [5,19] are often sensitive to parameter selection and lack robustness in non-Gaussian noise environments, limiting their applicability in low-power IoT and UAV signal processing tasks.

Recent developments in deep learning, particularly convolutional neural networks (CNNs) and generative adversarial networks (GANs), have demonstrated superior performance in complex signal detection and denoising tasks [20]. These approaches offer enhanced feature extraction and noise resilience, enabling more accurate and intelligent identification of LoRa signals in challenging electromagnetic environments. However, systematic theoretical analysis and engineering implementation of protocol-level jamming mechanisms, such as preamble injection, remain in their infancy.

While predictive jamming and energy depletion attacks have demonstrated the potential to disrupt LoRa-based UAV communications, they often suffer from limitations such as reliance on accurate traffic prediction, high energy consumption, or delayed disruption effects. In contrast, the preamble injection technique proposed in this work offers several distinct advantages. By targeting the critical synchronization phase of LoRa packet reception, our method enables rapid and real-time interference, leading to immediate communication failure. Moreover, because the interference is precisely timed and focused, it achieves a high disruption accuracy with minimal energy expenditure. These characteristics make preamble injection particularly suitable for scenarios requiring fast, efficient, and reliable UAV countermeasures, and highlight the practical significance and novelty of our approach compared to existing methods.

## 3. Methods

**Figure 1:**
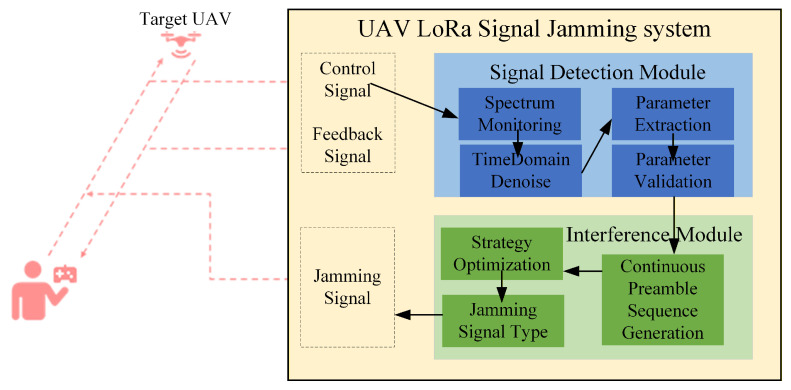
LoRa Communication Link Jamming Framework.

The main functions of the jamming technique are illustrated in Figure 1, which is composed of four main functional modules: signal monitoring, parameter analysis, interference signal generation, and interference signal injection. Initially, the signal monitoring module continuously scans the designated frequency bands to detect and capture LoRa signals of interest. Once a target signal is identified, the parameter analysis module performs reverse engineering to extract key modulation parameters, including center frequency, bandwidth, spreading factor, and preamble structure. These extracted parameters are then utilized by the interference signal generation module to synthesize customized interference signals, such as pseudo-spreading or synchronized frequency-hopping signals, which are specifically tailored to match or disrupt the target LoRa communication. Finally, the interference signal injection module transmits the generated interference signals into the communication channel via a software-defined radio platform. This closed-loop workflow enables precise and adaptive interference against LoRa systems, significantly increasing the bit error rate and packet loss rate at the receiver, thereby validating the effectiveness of the proposed interference strategy.

### 3.1. Signal Extraction

The main challenges in LoRa signal feature extraction are as follows: First, LoRa signals employ spread spectrum modulation, resulting in low power spectral density. In complex electromagnetic environments, these signals are easily masked by noise and other signals, making traditional energy detection methods ineffective. Second, different LoRa symbols exhibit extremely similar energy characteristics in the time domain, lacking significant power peak variations. This renders energy-based methods for symbol boundary detection and period estimation invalid. Furthermore, the physical layer parameters of LoRa signals—such as spreading factor (SF), bandwidth (BW), and coding rate (CR)—are highly diverse and often unknown in practical applications, increasing the complexity of signal identification and demodulation. Finally, LoRa signals frequently operate under negative signal-to-noise ratio (SNR) conditions, where signal power is lower than noise power. Additionally, multipath fading, frequency offset, and clock drift introduce channel impairments that severely degrade the performance of traditional correlation detection and matched filtering methods. Therefore, more intelligent and robust signal processing techniques are required to achieve accurate feature extraction and parameter estimation.

To address these challenges, this paper leverages neural network technologies for practical signal extraction. By utilizing the powerful feature learning capabilities of CNNs, the limitations of traditional energy detection methods in LoRa signal detection are effectively overcome. Simultaneously, GANs are employed for denoising in the time domain, enabling precise feature extraction of LoRa signals under negative SNR conditions.

#### 3.1.1. Signal Perception Based on CNNs

SNR is a crucial parameter in the generation of training datasets for CNN-based models. Higher SNR values make LoRa signal features more prominent in spectrograms, thereby reducing the difficulty of recognition for CNN networks. To enhance the robustness of the trained CNN model, this study focuses on strengthening the neural network’s perception ability for LoRa signals under low SNR conditions. In low SNR environments, LoRa RF signal samples with varying spreading factors, bandwidths, and SNR levels are collected, and multiple types of typical interference are introduced to enrich the data distribution. The raw IQ signals are processed using short-time Fourier transform (STFT) or fast Fourier transform (FFT) and converted into two-dimensional spectrogram images as inputs to the network. The dataset also includes background noise samples and non-LoRa signals from different channel environments to improve the model’s adaptability to complex electromagnetic scenarios. The spectrogram images obtained through this approach provide a solid foundation for the training and testing of the CNN model.

**Figure 2:**
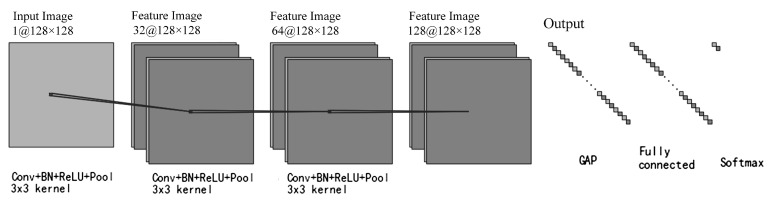
CNN structure for LoRa signal extraction.

To capture the spatial features of LoRa signal spectrograms, a CNN model consisting of multiple convolutional and pooling layers is constructed, as illustrated in Figure 2. Small convolution kernels are employed in the convolutional layers to extract fine-grained features of the signals, while pooling layers are utilized for dimensionality reduction and to enhance the robustness of the extracted features. After feature extraction, the network performs classification of signal presence through fully connected layers. This architecture effectively captures key features in the spectrograms while maintaining computational efficiency, making it suitable for real-time signal detection tasks on embedded platforms.

Compared with methods that use raw signal data [21], training on spectrogram images enables the model to efficiently identify key features in the signal’s frequency domain. This approach not only improves recognition accuracy but also reduces training time, making it highly suitable for practical interference detection scenarios.

#### 3.1.2. Signal Denoising Based on GANs

Although CNNs demonstrate effective perception of LoRa signals under low SNR conditions in experimental settings, signal propagation in practical scenarios is often subject to substantial noise interference, which can severely disrupt the structural information of wireless signals and significantly degrade signal sensing performance. Therefore, signal denoising is a crucial step in the extraction of target LoRa signals. Traditional signal denoising methods mainly rely on signal processing techniques such as wavelet transform (WT) [21,22] and empirical mode decomposition (EMD) [5,19]. While these methods perform well in handling linear or quasi-stationary noise, they suffer from strong parameter sensitivity, reliance on manual expertise, poor adaptability to non-Gaussian noise, and are generally incapable of preserving the temporal structural features of signals. As a result, their effectiveness is limited in applications such as low-power IoT and UAV signal processing tasks.

With the advancement of deep learning technologies and the expansion of their application domains, GANs demonstrate excellent performance in wireless signal denoising tasks. GANs can effectively restore fine-grained high-frequency details, thereby reducing blurring and excessive smoothing in the output signals. Moreover, the adversarial training mechanism of GANs ensures strong robustness under low SNR conditions, enabling the denoising model to maintain good generalization capabilities even in complex environments.

**Figure 3:**
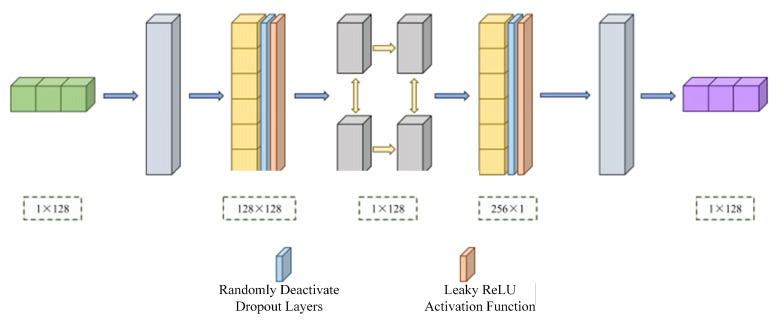
LR-GAN generator structure.

**Figure 4:**
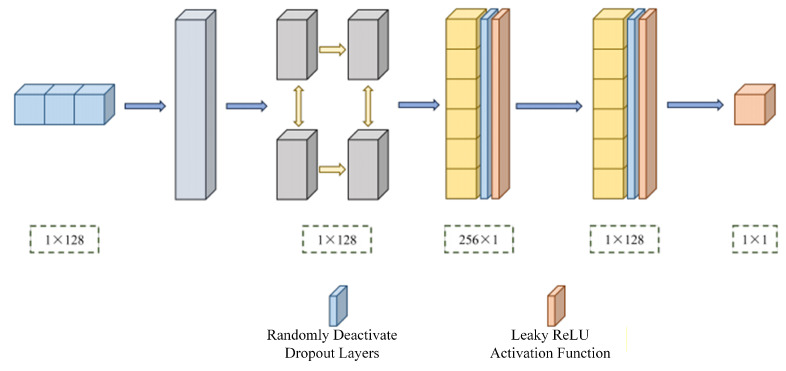
LR-GAN discriminator structure.

The traditional GAN architecture primarily consists of two components: a generator (G) and a discriminator (D), which learn the data distribution through alternating training [12]. To make the generated samples from GANs more closely resemble real data, an average discrimination strategy is adopted. In this approach, the discriminator no longer judges the authenticity of a single sample independently; instead, it compares the sample against the average of the generated samples. This forms the LR-GAN generator and discriminator architectures, as illustrated in Figure 3 and Figure 4, respectively.

The generator adopts a deep neural network architecture that integrates temporal modeling and nonlinear feature transformation. It mainly comprises normalization layers, fully connected layers, a Bi-LSTM network, activation functions, dropout layers, and a denormalization module, mapping the noise-corrupted raw IQ signals to noise-free signals that closely approximate the true distribution. The input to the generator network is a complex IQ sequence of length 128, represented as a 1 × 128 two-dimensional real-valued vector. The normalized signal is first passed through a fully connected layer, expanding it to a 128 × 128 feature representation, followed by a LeakyReLU activation function and a dropout layer, which enhance the model’s nonlinear modeling capability and reduce the risk of overfitting. Subsequently, a Bi-LSTM layer is employed to extract contextual information in the temporal dimension, enabling the network to capture symbol correlations and chirp characteristics inherent to LoRa signals. The output of the Bi-LSTM is further processed by another fully connected layer to extract higher-order temporal features. Finally, denormalization is applied to restore the signal amplitude to its original physical scale, and the generated output signal maintains the same shape as the original input.

The discriminator is composed of modules similar to those in the generator and employs a relative evaluation mechanism to determine whether the input sample is closer to the real data distribution than the average generated sample. The input to the discriminator network consists of IQ samples from both the generated and real signals, each with a length of 128. After normalization, the signals are sequentially processed through a fully connected layer, a Bi-LSTM network, and a nonlinear transformation module constructed with dropout layers and LeakyReLU activation functions. The final output is a 1 × 1 scalar value, which serves as a relative authenticity score for the input signal.

Signals denoised by GAN exhibit clearer modulation structures and symbol periods, facilitating subsequent parameter analysis and signal identification tasks. This approach provides robust technical support for high-precision feature extraction of LoRa signals in complex environments.

To evaluate the feasibility of deploying the proposed CNN and GAN models in real-time UAV communication scenarios, we analyzed both processing latency and hardware requirements. The CNN-based signal detection model was tested on an ARM Cortex-A53 embedded processor, achieving an average inference time of less than 10 ms per 128-sample input, which is sufficient for real-time LoRa signal monitoring. The GAN-based denoising model, designed with lightweight layers and optimized Bi-LSTM units, demonstrated an average processing time of approximately 20 ms per sequence on the same platform.

These results indicate that both models can meet the timing constraints of typical UAV remote control and telemetry applications. For even more resource-constrained environments, further optimizations such as model pruning, quantization, and hardware acceleration (e.g., using DSP or FPGA) can be explored to reduce latency and computational load. The current implementation provides a practical balance between performance and efficiency, supporting real-time operation on widely available embedded hardware.

### 3.2. Protocol Interference from Preamble Injection

To effectively interfere with perceived signals, it is necessary to compromise both anti-jamming mechanisms of LoRa signals: spread spectrum and frequency modulation. Spread spectrum extends the original signal over a wider frequency band using specific spreading codes, thereby enhancing its anti-jamming capability and concealment; frequency modulation, such as frequency hopping, disperses the signal energy by varying the carrier frequency, reducing the risk of interference at any single frequency point and improving communication reliability [8]. Once the characteristics of the target UAV communication protocol have been identified, similar interfering signals can be generated to disrupt both anti-jamming techniques accordingly.

For LoRa’s spread spectrum mechanism, interference can be achieved by generating pseudo spread-spectrum signals with identical or similar spreading parameters to the target signal. These signals are injected into the target frequency band using software-defined radio platforms (such as USRP), causing inter-code interference during the de-spreading process at the receiver and increasing the bit error rate, thereby enabling rapid jamming. For LoRa’s frequency modulation mechanism, interference signals can be generated by analyzing the target signal’s frequency distribution, modulation rate, and switching rules, and synchronizing the interference sequence with the target’s frequency modulation pattern to achieve continuous disruption of the communication link. Additionally, predictive modeling of the target’s frequency modulation behavior can be employed to pre-deploy interference signals, further improving the success rate of jamming.

Due to the proprietary nature of the LoRa physical layer protocol, Semtech has not publicly disclosed the complete physical layer modulation process. According to existing reverse engineering studies, such as those by Tapparel and Qolbuna [20,21], the typical physical layer encoding process of LoRa signals includes Hamming encoding, per-byte whitening, per-byte shuffling, interleaving, and Gray coding. Through the combination of these encoding procedures, the LoRa protocol provides reliable data transmission services, ensuring data integrity and security over wireless channels.

**Figure 5:**
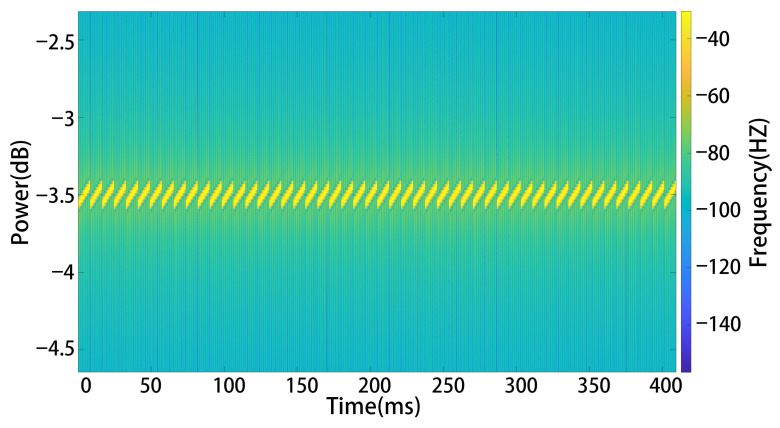
Analog interference signal spectrum.

The specific process for generating interference signals is as follows: First, input parameters are checked. The loramod function requires at least four input parameters: the symbol vector x, spreading factor (SF), signal bandwidth (BW), and sampling frequency (fs). The parameter Inv is optional and indicates whether the modulation uses upchirp or downchirp; the default is upchirp with linearly increasing frequency. The procedure then involves calculating the symbol duration and number of samples, computing frequency modulation-related parameters, determining instantaneous frequency and phase, and finally generating the corresponding IQ waveform, which is reshaped into a column vector y for output. In simulation experiments, the spectrogram of the simulated interference signal is shown in Figure 5.

## 4. Experiments

To validate the interference effectiveness of the jamming signal generation algorithm on LoRa communication systems, target perception and signal parameter extraction were performed on the target UAV in real-world scenarios and simulation experiments separately, followed by the injection of jamming signals. Quantitative experiments were conducted to evaluate the practical effectiveness and applicability of the proposed algorithm in actual application environments.

### 4.1. Setup

**Figure 6:**
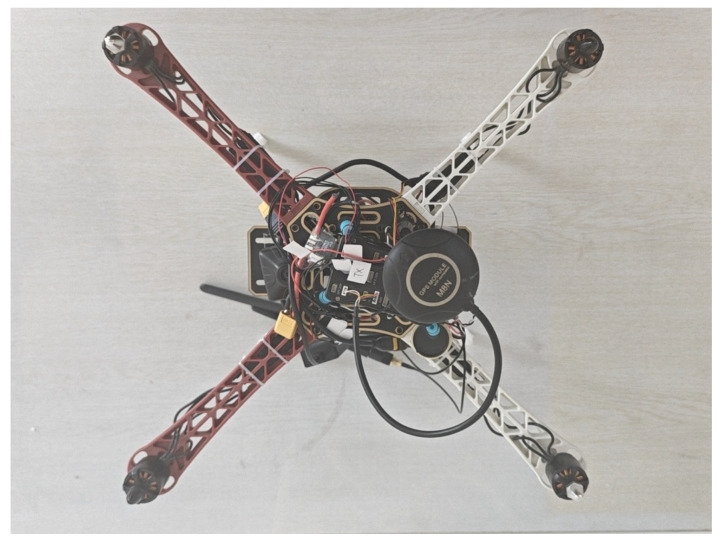
Experimental UAV.

**Figure 7:**
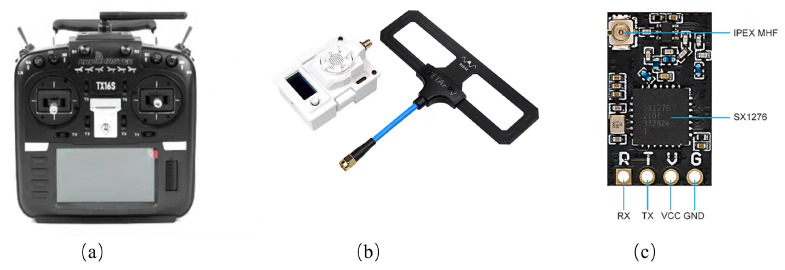
UAV remote control communication module. The three modules shown are: (**a**) Remote control, (**b**) RF signal transmitting module, and (**c**) RF signal pre-receiving module.

ExpressLRS was selected as the target UAV system in the experiment, paired with the Radiomaster TX16S MKII transmitter and the BETAFPV ELRS Micro RF module. The communication setup formed by these UAV and radio devices provides stable control signals, operates at a frequency of 915 MHz, supports a spreading factor range of 7–12, and allows for adjustable transmission power between 14 dBm and 20 dBm, meeting the power requirements of typical UAVs. Additionally, the racing drone is equipped with a BETAFPV ELRS Nano receiver featuring the SX1276 chip to receive signals from the transmitter. The experimental UAV and its remote communication modules are shown in Figure 6 and Figure 7, respectively.

**Figure 8:**
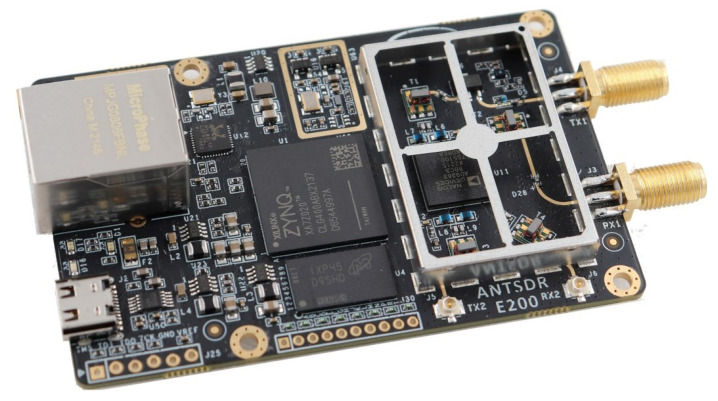
AntSDR E200 SDR RF front end.

For the generation and transmission of interference signals, GNU Radio was selected for prototype development, while SDR hardware support was provided by the USRP platform designed by Ettus Research. These components work together to enable the generation of interference signals. The AntSDR E200 was chosen as the front-end transmitter for emitting interference signals; it features dual channels for wireless signal transmission and reception, supports sampling rates from 200 kHz to 61.44 MHz, offers a tuning range covering 10 kHz to 6 GHz, and provides RF bandwidth from 200 kHz to 5.6 MHz with a maximum RF transmit power of up to 10 dBm, as shown in Figure 8.

It should be noted that the experiments in this study were primarily conducted on a single UAV system under controlled laboratory conditions. This approach was chosen to ensure the repeatability and accuracy of measurements, and to allow for detailed analysis of the proposed preamble injection jamming method without interference from external variables. While this setup provides a clear demonstration of the technical feasibility and effectiveness of our method, we acknowledge that real-world UAV deployments often involve multiple UAVs, diverse LoRaWAN network configurations, and more complex electromagnetic environments.

Future work will extend the experimental scope to include multi-UAV collaborative scenarios, various LoRaWAN network topologies (such as multi-gateway and distributed node deployments), and real-world interference conditions. These additional tests will further validate the robustness and generalizability of the proposed jamming technique. The current focus on a single UAV system serves as a necessary first step toward establishing a controlled baseline for subsequent large-scale and more realistic evaluations.

### 4.2. Indicators of Interference Effect Evaluation

To evaluate the communication suppression capability and energy efficiency advantages of the continuous preamble interference method, three key performance metrics were designed. These metrics compare and analyze the effects of continuous preamble interference versus traditional white noise interference from three perspectives: communication link quality, modulation and demodulation accuracy, and interference power efficiency.

**Packet Reception Rate (PRR)** reflects the proportion of data packets successfully received and correctly demodulated by the receiver within a unit time. It serves as a fundamental indicator for assessing the effectiveness of the communication link and is defined as:

(1) $$PRR=\frac{N_{recv}}{N_{sent}}\times 100\%$$

where $N_{recv}$ denotes the number of successfully received data frames and $N_{sent}$ represents the total number of transmitted packets. *PRR* indicates the impact of different interference methods on the availability of the communication system.

**Symbol Error Rate (SER)** measures the proportion of symbol decision errors occurring during the modulation and demodulation process, reflecting the extent to which interference disrupts demodulation. This metric is particularly suitable for analyzing signal-level interference characteristics and is defined as:

(2) $$SER=\frac{N_{ErrorSym}}{N_{TotalSym}}\times 100\%$$

where $N_{ErrorSym}$ represents the number of erroneously detected symbols, and $N_{TotalSym}$ denotes the total number of received symbols. In this experiment, SER is used to quantify the suppression capability of the interference signal on the recognition accuracy of the received signal.

To further assess the efficiency differences in energy consumption between various interference methods, this study introduces the **Equivalent Jamming Power Difference (EJPD)**. EJPD measures the minimum transmission power required to achieve a fixed interference effect and expresses the difference between two interference methods in logarithmic form, defined as:

(3) $$EJPD=\frac{A_{noise}}{A_{chirp}}\times 100\%$$

where $A_{noise}$ and $A_{chirp}$ represent the minimum transmission power (in dBm) of white noise interference and continuous preamble interference, respectively, required to reduce the PRR of the target LoRa communication link to nearly zero. A larger EJPD indicates that the noise-based jammer requires significantly higher power than the preamble-based jammer to achieve the same PRR reduction.

### 4.3. CNN and GAN Models Construction

Both CNN and GAN models were trained using a learning rate of 0.001. The dataset consisted of 10,000 samples, randomly divided into 80% for training and 20% for validation. Each model was trained for 30 epochs with a batch size of 64. These parameters were selected to achieve a balance between training efficiency and model performance.

Raw IQ signal data were collected and segmented into fixed-length frames. Each frame was converted into a frequency-domain representation using the FFT. Spectrogram images were then generated from these FFT results. To ensure robustness, noise samples were added by mixing signal frames with random noise before FFT processing. All spectrogram images were normalized and resized to 64 × 64 pixels prior to input into the neural networks.

This pipeline allows for consistent and efficient training and evaluation of both the CNN and GAN models, facilitating accurate signal classification and generation in the presence of interference.

### 4.4. Single Frequency Band Interference Test

**Figure 9:**
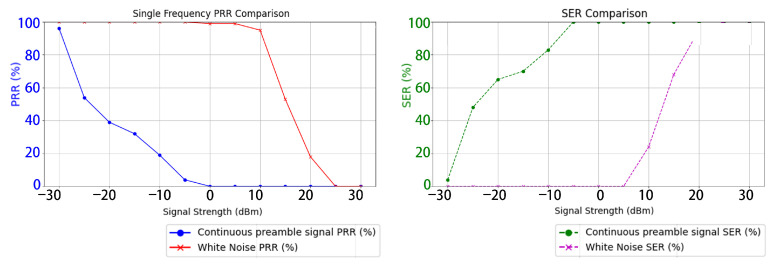
Evaluation of communication interference effects at a single frequency point.

In the single-frequency band interference test, the list of data packets to be transmitted between the two LoRa communication modules was predetermined prior to the experiment. Both modules initiated communication under interference conditions, transmitting a total of 1000 packets to evaluate the effects of interference at various power levels. The two sets of LoRa modules were subjected to interference using either continuous preamble signals or noise signals, with interference intensity ranging from −30 dBm to 30 dBm. Multiple repeated experiments were conducted to measure the variations in PER and SER under different noise interference conditions, as shown in Figure 9.

Experimental results indicate that as the interference signal strength increases, LoRa communication experiences varying degrees of disruption. Notably, continuous preamble interference can reduce the target signal’s PRR with lower power consumption and increase its SER, achieving optimal performance when the signal strength approaches −5 dBm(transmitter output power). In contrast, traditional noise interference only begins to affect the target when the signal strength exceeds 5 dBm, and complete disruption of the target signal is achieved only at an interference strength of 25 dBm.

**Figure 10:**
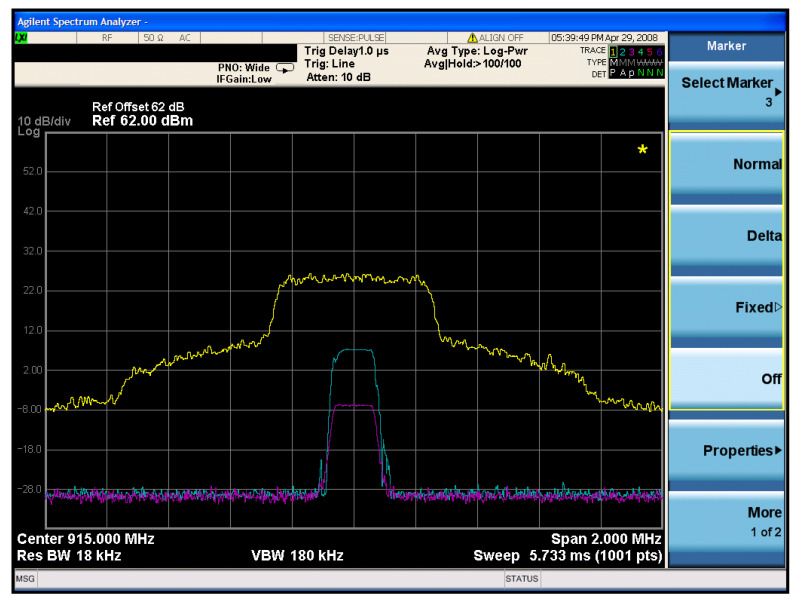
Comparison of signal source transmit power when PRR of a single frequency is 0.

When both types of interference signals were used to reduce the target communication PRR to zero, the signal strength was recorded using a spectrum analyzer(SA), as illustrated in Figure 10. Under this condition, the LoRa communication signal power was −7 dBm, the preamble interference signal power was 7 dBm, and the white noise signal power was 24 dBm. The calculated equivalent jamming power ratio was 50.1%.

**Figure 11:**
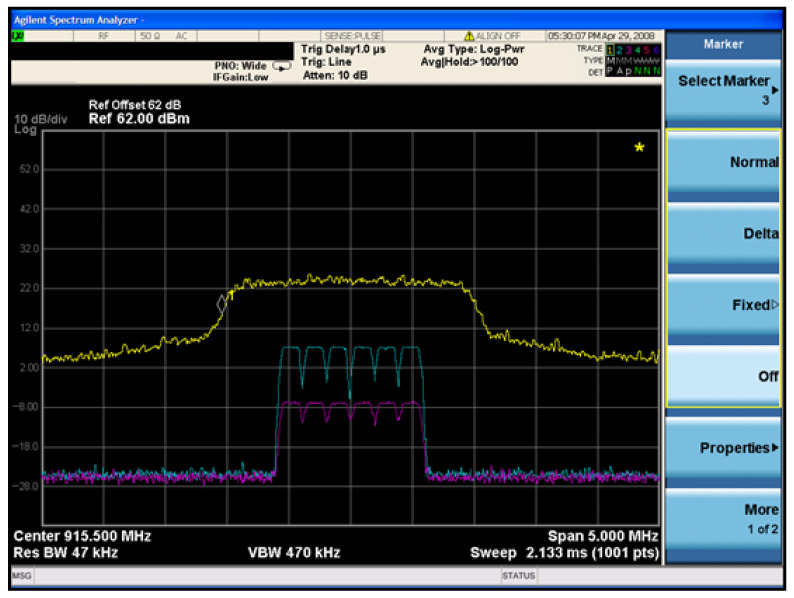
Comparison of signal source transmit power when frequency hopping PRR is 0.

Note that the transmit power levels stated (e.g., 24 dBm for white noise transmission) were achieved using an external RF power amplifier connected to the AntSDR E200 module, whose internal maximum output is 10 dBm. For the SA measurements shown in Figure 10 and Figure 11, the SDR output was directly connected to the SA input. The “dBm” values on the SA traces represent power spectral density per frequency bin, not total signal power. Actual total power should be integrated over the signal bandwidth, but the relative differences in power spectral density between the communication signal, preamble interference, and white noise signals remain valid for the analysis and conclusions.

### 4.5. Frequency Hopping Interference Test

**Figure 12:**
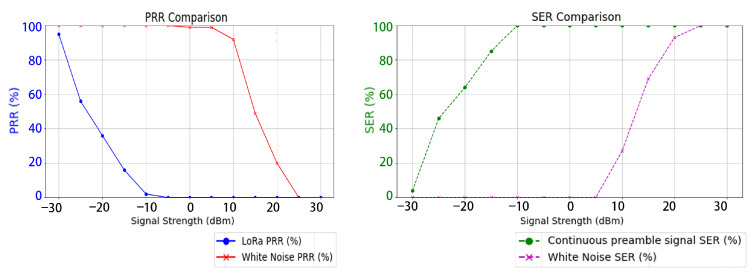
Evaluation of interference effects under frequency-hopping communication.

In the frequency-hopping interference test, the LoRa communication modules were configured to randomly hop among six central frequencies, transmitting a total of 1000 data packets. The PRR and SER were recorded for each frequency band, as illustrated in Figure 12. Experimental results show that as the interference signal strength increases, LoRa communication experiences varying levels of disruption. Continuous preamble interference was able to reduce the target signal’s PRR and increase its SER with lower power consumption, achieving optimal performance at a signal strength of approximately −10 dBm. In contrast, traditional noise interference only began to affect the target when the signal strength exceeded 10 dBm, and complete disruption was achieved at 25 dBm.

When both types of interference signals were used to reduce the target communication PRR to zero, the signal strength was recorded using the SA, as shown in Figure 11. Under these conditions, the LoRa communication signal power was −7 dBm, the preamble interference signal power was 7 dBm, and the white noise signal power was 24 dBm. The equivalent jamming power ratio was consistent with that observed in the single-frequency band scenario.

All interference experiments described in Section 4.3 and Section 4.4 were conducted in a simplified shielded chamber, where the positions of the LoRa transmitting node, receiving node, and the jammer transmitting antenna were fixed throughout the tests. The only variable in these experiments was the design of the signal source format. The background noise level in the chamber was maintained below −90 dBm.

During testing, both the custom pilot-correlated noise signal and the white noise signal were transmitted using the same antenna, with its position unchanged between experiments; only the signal source was switched. Therefore, the present study focuses exclusively on the impact of signal format on the required jamming power. Factors related to antenna directivity, gain, polarization, and radiation efficiency—known to significantly influence real-world jamming effectiveness—were intentionally excluded by using the same transmitting antenna for all tests. The effects of antenna orientation on the signal-to-interference-plus-noise ratio (SINR) were not investigated in this work, but should be considered in future field applications.

### 4.6. Drone Remote Control Signal Interference Test

**Figure 13:**
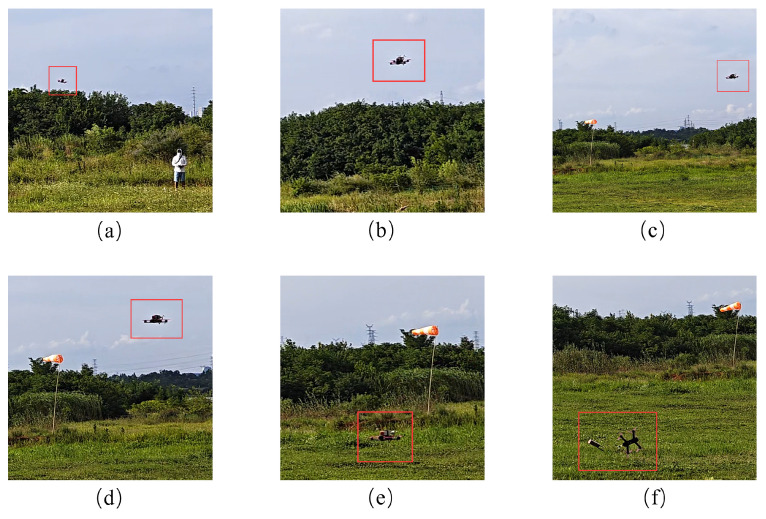
Field test results: (**a**) UAV takeoffs, (**b**,**c**) UAV in flight, (**d**) UAN jamming, (**e**) UAV approaches the ground, and (**f**) UAV crashes.

To validate the practical effectiveness of the preamble injection jamming method, UAV remote control signal interference tests were conducted in an open environment. During testing, the communication distance between the UAV and the remote controller was set to approximately 500 m, with the UAV, controller, and jammer positioned nearly in a straight line. The interference signal transmission power was set to 50 W, and both continuous preamble interference signals and white noise signals were evaluated. The experimental results are presented in Figure 13. The red boxes in Figure 13 are the identifications of the test UAV.

Experimental results demonstrate that white noise interference begins to effectively disrupt the UAV remote control link when the jammer is approximately 200 m away from the UAV. In contrast, the continuous preamble interference signal remains effective at distances up to 1000 m.

### 4.7. Comparison with Other Methods

To further demonstrate the effectiveness of our proposed preamble injection jamming method, we compare it with two representative approaches from recent literatures: the predictive jamming [2], the replay jamming [3], and the selective inference jamming [4]. All methods are evaluated under identical LoRaWAN communication settings and controlled experimental conditions, which is showned in Table 1.

The experimental results presented in Table 1 demonstrate the superior performance of our proposed preamble injection jamming method compared to predictive jamming, replay jamming, and selective jamming approaches. Specifically, our method achieves a significantly lower PRR of 5% and a higher SER of 92%, indicating a more effective disruption of LoRaWAN communications. Furthermore, the minimum effective jamming power required by our method is only −7 dBm, which is substantially lower than that of the other methods. In contrast, predictive jamming, replay jamming, and selective jamming methods exhibit higher PRR values (27%, 33%, and 20%, respectively) and lower SER values (65%, 56%, and 74%, respectively), signifying less efficient interference. Additionally, these methods require greater jamming power (10 dBm, 13 dBm, and 12 dBm, respectively) to achieve their effects, resulting in higher energy consumption.

### 4.8. Ethical and Regulatory Discussion

All experiments and jamming techniques described in this study were conducted in accordance with local governmental laws and regulations. The experimental procedures were performed under strictly controlled laboratory conditions, with all necessary permissions and approvals obtained prior to testing.

## 5. Discussion

In the frequency-hopping interference test (Section 4.4), we selected six random frequencies within a relatively narrow band. When attempting to extend the hopping scheme to more than ten frequency channels, we observed that it was not possible to maintain uniform transmission power across all channels. This limitation may be attributed to the transmitter hardware or the algorithmic model, and is currently under further investigation.

The primary focus of this experiment was to evaluate the effectiveness of spread spectrum anti-jamming techniques. For the hopping parameters, we used SF = 7 and BW = 125 kHz with N = 8 channels, resulting in a practical hopping interval of approximately 8ms. The hopping rate in our tests was therefore about 125 Hz.

It should be noted that the merits of the proposed technique may vary when applied to a significantly broader frequency region or higher hopping rates, as hardware and processing constraints could affect jamming performance. Further optimization and investigation are required to ensure consistent effectiveness under such conditions.

## 6. Conclusions

This paper addresses the challenges of low-altitude security and communication safety arising from the widespread deployment of highly maneuverable UAVs in recent years. Focusing on the commonly used LoRa communication protocol in UAVs, we propose an adaptive jamming method that integrates neural network-based signal detection, reverse parameter extraction, and the preamble injection jamming method. Efficient detection and precise denoising of LoRa signals in complex electromagnetic environments are achieved using CNNs and GANs, providing a solid foundation for subsequent parameter analysis and interference signal generation. By combining protocol-level preamble injection techniques, the method effectively disrupts the synchronization and demodulation processes of UAV communication links, significantly improving jamming efficiency, reducing energy consumption, and extending the effective jamming range. Experimental results demonstrate that the proposed approach outperforms traditional physical layer jamming methods in terms of signal detection accuracy and jamming energy efficiency, offering practical advantages of low cost and high performance. This research presents a novel and intelligent technical pathway for low-altitude security and UAV information countermeasures, and is of significant importance for enhancing the precision and practicality of UAV communication countermeasures.

## Figures and Tables

**Table 1:** Comparison with other methods.

Method	PRR	SER	Min.EP
**Ours Method**	5%	92%	−7 dBm
Prejamming	27%	65%	10 dBm
Rejamming	33%	56%	13 dBm
Selejamming	20%	74%	12 dBm

## Data Availability

The data that support the findings of this study are available from the corresponding author upon reasonable request.
